# Supplementary material for: The association between caesarean section delivery and later life obesity in 21-24 year olds in an Urban South African birth cohort
Source: PLoS One. 2019 Nov 14;14(11):e0221379. doi: 10.1371/journal.pone.0221379 (PMC6855451; doi:10.1371/journal.pone.0221379)
Supplement: S1 Stata code — (PDF) [file pone.0221379.s001.pdf]

S1 Stata code. Showing heterogeneity in obesity rates between male and female young adults

```
. mi estimate, irr

Multiple-imputation estimates                   Imputations       =         20
Poisson regression                              Number of obs     =        889
                                                Average RVI       =     0.0076
                                                Largest FMI       =     0.0484
DF adjustment:   Large sample                   DF:     min       =   8,192.03
                                                        avg       =   1.05e+10
                                                        max       =   2.62e+10
Model F test:       Equal FMI                   F(   6, 1.4e+06)  =      10.57
Within VCE type:       Robust                   Prob > F          =     0.0000
```

ob_non	IRR	Std. Err.	t	P>|t|	[95% Conf.	Interval]
mod						
assisted VD	1.406908	.6511281	0.74	0.461	.567967	3.485046
C/section	1.646095	.4085239	2.01	0.045	1.012059	2.677343
gender						
Female	7.648986	2.233071	6.97	0.000	4.316197	13.55522
BWeight	1.000445	.0002003	2.22	0.026	1.000052	1.000837
parity	1.014772	.0609171	0.24	0.807	.9021333	1.141475
mothersedu2	1.197227	.3951255	0.55	0.585	.6269163	2.286354
_cons	.0062771	.0047272	-6.73	0.000	.0014346	.0274655
