# Supplementary material for: The association between caesarean section delivery and later life obesity in 21-24 year olds in an Urban South African birth cohort
Source: PLoS One. 2019 Nov 14;14(11):e0221379. doi: 10.1371/journal.pone.0221379 (PMC6855451; doi:10.1371/journal.pone.0221379)
Supplement: S2 Stata code — (PDF) [file pone.0221379.s002.pdf]

S2 Stata code. Comparing the mean of the observed and imputed data

```
[illegible] .
22 . mi xeq 1/2: summarize BWeight if Mis_BWeight==0; ///
   > summarize  BWeight if Mis_BWeight==1; ///
   > summarize  BWeight

   m=1 data:
   -> summarize BWeight if Mis_BWeight==0

       Variable |        Obs        Mean    Std. Dev.       Min        Max
   -------------+---------------------------------------------------------
        BWeight |        888    3085.562    468.5039       1125       4920
   -> summarize  BWeight if Mis_BWeight==1

       Variable |        Obs        Mean    Std. Dev.       Min        Max
   -------------+---------------------------------------------------------
        BWeight |          1    3215.576           .   3215.576   3215.576
   -> summarize  BWeight

       Variable |        Obs        Mean    Std. Dev.       Min        Max
   -------------+---------------------------------------------------------
        BWeight |        889    3085.708    468.2603       1125       4920

   m=2 data:
   -> summarize BWeight if Mis_BWeight==0

       Variable |        Obs        Mean    Std. Dev.       Min        Max
   -------------+---------------------------------------------------------
        BWeight |        888    3085.562    468.5039       1125       4920
   -> summarize  BWeight if Mis_BWeight==1

       Variable |        Obs        Mean    Std. Dev.       Min        Max
   -------------+---------------------------------------------------------
        BWeight |          1    3588.146           .   3588.146   3588.146
   -> summarize  BWeight

       Variable |        Obs        Mean    Std. Dev.       Min        Max
   -------------+---------------------------------------------------------
        BWeight |        889    3086.127    468.5433       1125       4920

23 .
24 . mi xeq 1/2: sum mothersedu2 if Mis_mothersedu2==0; ///
   > sum mothersedu2 if Mis_mothersedu2==1; ///
   > sum mothersedu2

   m=1 data:
   -> sum mothersedu2 if Mis_mothersedu2==0

       Variable |        Obs        Mean    Std. Dev.       Min        Max
   -------------+---------------------------------------------------------
    mothersedu2 |        858    .0780886    .2684674          0          1
   -> sum mothersedu2 if Mis_mothersedu2==1

       Variable |        Obs        Mean    Std. Dev.       Min        Max
   -------------+---------------------------------------------------------
    mothersedu2 |         31    .0549069    .2611283  -.5407107   .5958778
   -> sum mothersedu2
```

Variable	Obs	Mean	Std. Dev.	Min	Max
mothersedu2	889	.0772802	.2681052	-.5407107	1

```
m=2 data:
-> sum mothersedu2 if Mis_mothersedu2==0
```

Variable	Obs	Mean	Std. Dev.	Min	Max
mothersedu2	858	.0780886	.2684674	0	1

```
-> sum mothersedu2 if Mis_mothersedu2==1
```

Variable	Obs	Mean	Std. Dev.	Min	Max
mothersedu2	31	.0959817	.2477908	-.4441501	.4968701

```
-> sum mothersedu2
```

Variable	Obs	Mean	Std. Dev.	Min	Max
mothersedu2	889	.0787125	.2676635	-.4441501	1
