# Supplementary material for: The association between caesarean section delivery and later life obesity in 21-24 year olds in an Urban South African birth cohort
Source: PLoS One. 2019 Nov 14;14(11):e0221379. doi: 10.1371/journal.pone.0221379 (PMC6855451; doi:10.1371/journal.pone.0221379)
Supplement: S1 Table — (PDF) [file pone.0221379.s003.pdf]

S1 Table. Body mass index categories of study participants by socio-demographic characteristics

	Body Mass Index categories										
	Total N=889		Underweight n=95		Normal BMI n=513		Overweight n=175		Obese n=106		P value
	n	%	n	%	n	%	n	%	n	%	
**Young adult characteristic**											
Sex											<0.001
Male	444	49.9	64	67.4	312	60.8	55	31.4	13	12.3	
Female	445	50.1	32	32.6	201	39.2	120	68.6	93	87.7	
Ethnicity											0.039
Black	803	90.3	78	82.1	468	91.2	161	92.0	96	90.6	
Others	86	9.7	18	17.9	45	8.8	14	8.0	10	9.4	
Alcohol intake											0.527
No drinking	560	63.0	23	24.2	165	32.2	46	26.3	30	28.3	
Yes drinking	264	29.7	58	61.1	321	62.6	115	65.7	66	62.6	
Unknown	65	7.3	14	14.7	27	5.3	14	8.0	10	9.4	
Smoking											<0.001
Non smoker	530	59.6	46	48.4	285	55.6	124	70.9	75	70.8	
Smoker	359	40.4	49	51.6	228	44.4	51	29.1	31	29.3	
Education											<0.001
<grade 12	349	39.3	51	53.7	213	41.5	49	28.0	36	34.0	
Completed grade 12	530	59.6	44	45.3	293	57.1	125	71.4	69	65.1	
Unknown	10	1.1	1.1	10.0	7	1.4	1	0.6	1	0.9	
**Early and maternal characteristic**											
Mothers' post-school education											0.756
No	792	92.2	88	92.6	456	92.6	154	88.0	93	87.7	
Yes	67	7.8	5	5.3	38	5.3	15	8.6	9	8.5	
Unknown	30	3.4	2	2.1	19	2.0	6	3.4	4	3.8	
Birth weight (kg)											0.550 [f]
LBW (<2.5)	74	8.3	7	7.4	43	8.4	19	10.9	5	4.7	
'normal' (2.5-4.0)	798	89.8	87	11.0	461	89.9	152	86.9	98	92.5	
Macrosomia (> 4)	16	1.8	1	1.1	8	1.6	4	2.3	3	2.8	
Unknown	1	0.1		-	1	0.19	0	-	0	-	
Birth weight (centile)											0.739
SGA (≤10th)	79	8.9	11	11.6	43	8.4	19	10.9	6	5.7	
AGA (≥10th-≤90th)	680	76.5	76	79.0	393	76.6	130	74.3	82	77.4	
LGA (>90th)	129	14.5	9	9.5	76	14.8	26	14.9	18	17.0	
Unknown	1	0.1	0	-	1	0.19	0	-	0	-	

Others - Indians and Coloured, BMI - body mass index, LBW - low birth weight, SGA - small for gestational age, AGA - appropriate for gestational age; LGA - large for gestational age,
P values <0.05 were considered statistically significant; Pearson's Chi-square test or Fisher's exact (f)
