# Supplementary material for: The association between caesarean section delivery and later life obesity in 21-24 year olds in an Urban South African birth cohort
Source: PLoS One. 2019 Nov 14;14(11):e0221379. doi: 10.1371/journal.pone.0221379 (PMC6855451; doi:10.1371/journal.pone.0221379)
Supplement: S2 Table — (PDF) [file pone.0221379.s004.pdf]

S2 Table. Body mass index categories of study participants by maternal characteristics (continuous)

			Body Mass Index (BMI) Category								
	Total		Underweight n=95		Normal BMI n=513		Overweight n=175		Obese n=106		P value
	Med	IQR	Med	IQR	Med	IQR	Med	IQR	Med	IQR	
Gestational age (weeks) at YA birth	38.0	38.0 – 39.0	38.0	38.0-39.0	38.0	38.0-38.0	38.0	38.0-39.0	38.0	37.0-40.0	0.772
Age at YA birth (years)	24.0	20.0 – 30.0	25.0	21.5-30.0	24.0	20.0-29.0	24.0	20.0-30.0	25.5	21.0-31.0	0.280
Parity at YA birth	2.0	1.0 – 3.0	2.0	1.0- 3.0	2.0	1.0- 3.0	2.0	1.0- 3.0	2.0	1.0- 3.0	0.019
Breastfeeding duration at infancy (months)	8.0	1.0 – 20.0	4.0	2.0-19.0	8.0	0.7-20.0	9.0	1.0-19.0	11.8	2.0-19.0	0.675

Med - median, IQR- interquartile range, BMI - body mass index
P values <0.05 were considered statistically significant; Kruskal Wallis
