# Supplementary material for: The association between caesarean section delivery and later life obesity in 21-24 year olds in an Urban South African birth cohort
Source: PLoS One. 2019 Nov 14;14(11):e0221379. doi: 10.1371/journal.pone.0221379 (PMC6855451; doi:10.1371/journal.pone.0221379)
Supplement: S3 Table — (PDF) [file pone.0221379.s005.pdf]

S3 Table. The association between mode of delivery and early adulthood obesity, stratified by sex (using normal BMI as reference*)* – complete case analysis

Variable	n	adjIRR	95%CI	P value
Main analysis				
NVD	84	1.00	reference	
AVD	4	1.51	0.62 – 3.73	0.368
CS	14	1.75	1.01 – 2.68	0.025
Male				
NVD	9	1.00	reference	
AVD	1	4.90	0.65 – 37.17	0.124
CS	3	4.01	1.14 – 14.09	0.031
Female				
NVD	79	1.00	reference	
AVD	3	1.12	0.41 – 3.07	0.717
CS	11	1.44	0.85 – 2.44	0.173

N=857; Poisson regression
OR – odds ratio, CI – confidence interval, NVD/AVD – Normal/Assisted vaginal delivery, CS – caesarean section.
n_o – number of obese participants with outcome(s) in row group
Adjusted for YAs' sex and birth weight; mothers' parity and education at YA's birth in all models.
