# Supplementary material for: The association between caesarean section delivery and later life obesity in 21-24 year olds in an Urban South African birth cohort
Source: PLoS One. 2019 Nov 14;14(11):e0221379. doi: 10.1371/journal.pone.0221379 (PMC6855451; doi:10.1371/journal.pone.0221379)
Supplement: S4 Table — (PDF) [file pone.0221379.s006.pdf]

S4 Table. Sensitivity analysis: the association between mode of delivery and early adulthood obesity under different scenarios

	**AdjIRR**	**95% CI**	**P value**
Combined normal and			
assisted vaginal delivery			
VD (normal and assisted)	1.00	reference	
CS	1.63	1.00 – 2.64	0.049
**Controlling for confounders**			
Lifestyle and behavioural			
characteristics + main analysis			
NVD	1.00	reference	
AVD	1.43	0.59– 3.49	0.428
CS	1.65	1.01 – 2.68	0.044
Early life factors only			
NVD	1.00	reference	
AVD	1.48	0.64 – 3.42	0.356
CS	1.53	0.94 – 2.47	0.086
Covariates linear/categorical			
NVD	1.00	reference	
AVD	1.48	0.62 – 3.53	0.378
CS	1.62	0.99 – 2.65	0.054

N= 889; Poisson regression

OR – odds ratio, CI – confidence interval, NVD/AVD – Normal/Assisted vaginal delivery

Adjustment for confounders

i. Combined NVD and AVD – adjusted for YA’s sex and birth weight; mothers’ parity, age, gestational age, and education at YA’s birth.

ii. Covariates linear/categorical – adjusted for YA’s birth weight and breastfeeding duration at infancy (categorical); mother’s parity and age at YA’s birth (categorical) + other covariates in main analysis.

iii. Lifestyle and behavioural characteristics – adjusted for YA’s breastfeeding duration during infancy, smoking habit, and alcohol intake + other covariates in main analysis.

Early life factors only – adjusted for YA’s sex, birth weight and breastfeeding duration at infancy; mothers’ parity, gestational age, age and education at YA’s birth.
